# The Effect of Implementing COVID-19 Infection Control Precautions on Conducting Simulation-Based Training Activities

**DOI:** 10.7759/cureus.23178

**Published:** 2022-03-15

**Authors:** Ghadah A Almujlli, Abdulmajeed S Alghosen, Maad F Alsaati

**Affiliations:** 1 Simulation and Skills Development Center, Princess Nourah Bint Abdulrahman University, Riyadh, SAU; 2 King Abduallah bin Abdulaziz University Hospital, Princess Nourah Bint Abdulrahman University, Riyadh, SAU

**Keywords:** medical education, learner evaluation, infection control protocol, covid-19, simulation-based training

## Abstract

Introduction

Due to the coronavirus disease 2019 (COVID-19) pandemic, onsite simulation training required infection control precautions. This study aimed to investigate the effect of implementing the infection control protocol on the participants' evaluation of simulation activities.

Materials and methods

The study included undergraduate female students from healthcare colleges in Princess Nourah University (PNU) who have attended simulation training events in the simulation and skills development center (SSDC). The study design was a cohort retrospective where data were retrieved from the SSDC participant evaluation database. The data included information related to participants' characteristics, simulation activities type, and learners' evaluation. The simulation activities included in the study were procedural simulation (PS) and full simulation (FS).

Results

The study included 837 subjects that were randomly chosen from January 2019 to December 2021. All participant's evaluations of simulation events conducted in the SSDC during the specified period were reviewed and randomly selected to be included in the study. Due to the COVID-19 pandemic, the activities were conducted under infection control protocol measures. There was a significant difference in evaluation results of FS and PS activities before and after implementing the COVID-19 infection control protocol (*p*-value < 0.001).

Conclusion

The study showed that conducting simulation activities in a simulation center under the infection control protocol does not negatively affect participants' evaluation of simulation events.

## Introduction

Simulation-based healthcare education has grown very noticeably over the past 10 years. The growth of simulation has presented a new direction in which the simulation was no longer seen as novel and needed to be justified or defended [1]. Clinical simulation has been defined as “A technique that creates a situation or environment to allow persons to experience a representation of a real event to enable effective collaboration and improve health outcomes” [2]. Simulation applications can be conducted for individuals with different experience levels, from students in the early years of medical schools to physicians with demonstrated years of experience. Thus, simulation is considered a lifelong educational tool for healthcare workers [3]. Consequently, studies have shown that utilizing simulation during pandemics to prepare and train healthcare practitioners is a very effective and efficient strategy [4-6].

Since the start of the coronavirus disease 2019 (COVID-19) pandemic, healthcare organizations around the globe have faced many challenges. One of those challenges was accommodating large numbers of infected patients given the limited resources such as not having well-trained healthcare practitioners who can tackle the challenges while dealing with COVID-19 patients. In essence, the need to train healthcare workers to deal with COVID-19 patients increased dramatically, and healthcare organizations sought adequate training, especially for the non-critical healthcare practitioners [7-8].

Due to the COVID-19 pandemic, simulation training requires special precautions to ensure a safe learning environment [9]. However, the effect of implementing restrictions on the learners’ perception and attitude towards training has not yet been explored. Therefore, the Simulation and Skills Development Center (SSDC) at Princess Nourah Bint Abdul Rahman University (PNU) has adopted an infection control protocol during onsite simulation activities. Therefore, this study aimed to describe the COVID-19 infection control protocol developed and implemented in SSDC and investigate participants’ attitudes and perceptions in simulation activities after implementing the infection control protocol.

## Materials and methods

This study was a retrospective study design, where data were retrieved from the SSDC database. The data included participants’ characteristics: student affiliation, healthcare college, and learners’ level. The data also included the learner’s evaluation of the simulation activities. The simulation activities included in the study were Procedural simulation activities (PS) and full simulation activities (FS).

FS is a terminology that SSDC has adapted from the “Simulation Scenario” definition to define an activity that includes specific learning objectives, scenario narrative, critical actions, debriefing guide, simulation setting, and, if applicable, instruction guide for the standardized patient (SP) [10]. PS is a simulation activity that uses a simulation modality, such as a task trainer, to complete the learning process of a specific skill or competency [2].

The study participants included were undergraduate students from five healthcare colleges in PNU, college of health and rehabilitation sciences, college of dentistry, college of medicine, college of nursing, and college of pharmacy. The participants were all female since Princess Nourah University is a women’s college. Subjects were randomly chosen from the SSDC learner evaluation database from January 2019 to December 2020. The number of subjects needed for the study was 500 participants. This sample size was required to detect a difference of proportion of 30% between groups and power = 85%. Out of 1503 participant evaluations in the database, 837 were randomly selected by a random number generator website [11]. The participants’ evaluations were collected through the two years; however, they are not for the same students, as each year has a new batch and they were not tracked. Therefore, this study examined discrete evaluations. The response rate for the SSDC participants’ evaluation ranged from 60-80% per activity. Participants’ evaluations were divided into two groups based on the time of conducting the event; the first group included participants’ evaluations of simulation activities conducted before the COVID-19 pandemic. The second group had an evaluation of simulation activities conducted during the COVID-19 pandemic.

SSDC collects participants’ feedback after every simulation activity. This process is done as part of the Society of Simulation in Healthcare (SSH) accreditation standards and ensures ongoing improvement of educational activities. SSDC collects evaluation results of simulation activities conducted in the center via an online form. The data collected included details related to the simulation activity: event name, event time, level of learners, learner’s affiliation, instructor name, instructor affiliation, etc. The participants could easily access the evaluation form via its QR code distributed throughout the SSDC.

To collect the learners’ evaluation of the simulation activities, the SSDC utilizes different instruments to evaluate the activities conducted in the center. Each evaluation form has a section that identifies information related to the event name, participant’s affiliation, and level, followed by the evaluation questions. Debriefing Assessment for Simulation in Healthcare^©^ instrument (DASH^©^) was utilized to assess the debriefing and FS activities [12]. The tool has 10 items, and each item has a 1-5 rating scale; 1 = strongly disagree, 2 = disagree, 3 = neutral, 4 = agree, 5 = strongly agree, and the total score is 50 points. All educators responsible for conducting or facilitating the simulation events were either simulation educators from SSDC with a background and experience in simulation education or faculty members who have taken the faculty development program given by the academic program department in SSDC. This program is conducted annually for all new faculty who plan to conduct simulation activities in SSDC. The program is given by SSDC staff with experience and qualifications in simulation education (e.g., have a master’s in medical education, are certified healthcare simulation educators (CHSE), have a master’s in simulation education, etc.).

The evaluation tool for PS activities has been developed by the SSDC and was validated using face and content validation. Validation measures used were face and content validity by three certified healthcare simulation educators and one instructional design specialist. The instrument was piloted among participants in 2018, and that pool of subjects was used to test the reliability. Cronbach’s alpha score for the instrument was 0.988. The tool has 10 items in FS and eight items in PS, and each item has a 1-5 rating scale; 1 = strongly disagree, 2 = disagree, 3= neutral, 4 = agree, 5 = strongly agree, and the total score is 40 points. Table 1 shows the items included in the evaluation forms of FS and PS.

**Table 1:** Evaluation form of different simulation activities DASH®: Debriefing Assessment for Simulation in Healthcare^©^

Simulation activity	Evaluation Items
Full simulation (FS) (DASH® tool)	1. The instructor set the stage for an engaging learning experience (Introducing sim safe: basic, assumption, faction contract, confidentiality, and room orientation). 2. The instructor maintained an engaging context for learning. 3. The instructor structured the debriefing in an organized way. 4. The instructor provoked in-depth discussions that led me to reflect on my performance. 5. The instructor identified what I did well or poorly and why. 6. The instructor helped me see how to improve or how to sustain good performance. 7. The setting and simulators resembled real-life situations. 8. The facility equipment and simulators worked as expected. 9. The temperature, lighting, and space were suitable. 10. I’m willing to attend more simulation scenarios.
Procedural simulation (PS)	1. This activity improved my knowledge and skills 2. This activity provides an effective way of learning new skills. 3. This activity enhances my confidence level. 4. The instructor contributes to improving my skills. 5. The simulation methodology contributes to improving my skills level. 6. The venue is organized and prepared for the learning skills needed for my training. 7. I would like to learn more using simulation in my future training. 8. I am satisfied with the level of clinical skills training.

Infection control protocol

**Figure 1:**
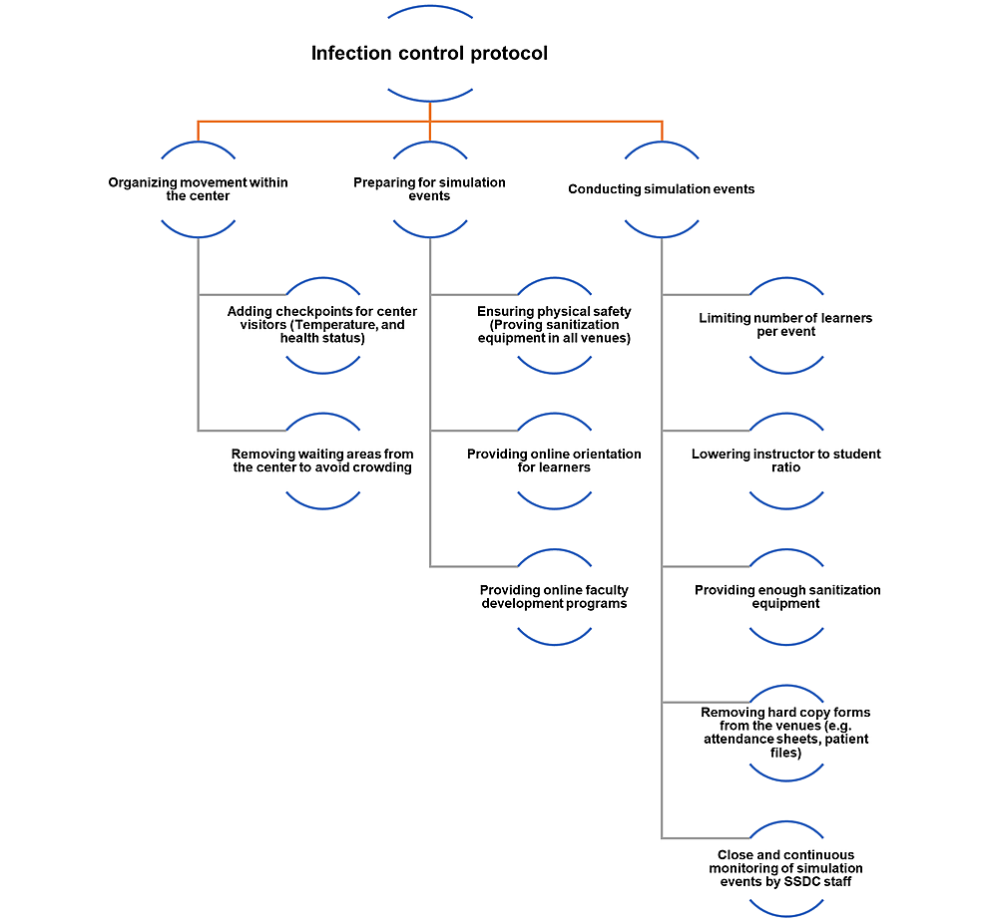
Summary of the critical areas of the SSDC infection control protocol SSDC: Simulation and Skills Development Center

The COVID-19 Infection control protocol was adopted by the Academic Program Department in SSDC and was approved by three SSDC staff members with nursing and pharmacy backgrounds. The infection control protocol was adapted from different references. The measures implemented during simulation activities were approved by a member from the COVID-19 task force that included different specialties such as medicine and nursing staff. The SSDC infection control protocol was reviewed after implementation by the Infection Control department in the university's hospital, King Abdullah bin Abdulaziz University Hospital (KAAUH). The developed protocol included the precautions taken before the simulation activities, during the simulation activity, and after finishing the activity. The protocol also included additional measures taken to ensure safety at facilities and create a safe learning environment. Figure 1 shows a summary of the critical areas of the SSDC infection control protocol.

SSDC has adapted the infection control protocol from several references, and the primary source was “SSSH guidelines for Clinical Simulation of Training and Education During and Beyond COVID-19 Pandemic,” which was developed by the Saudi Society for Simulation in Healthcare (SSSH) in 2020 [13]. The Academic Program Department adopted the guidelines in SSDC to develop an infection control protocol, and it was announced to SSDC staff. Simulation educators from the department and SSDC staff involved in the education activities implemented the protocol in the center, and it was also reported to all healthcare colleges.

A key aspect of checking learners, staff, and faculty attending the activities in SSDC were temperature checks at SSDC entry checkpoints and corresponding Tawakalna application [14]. The application is a governmental application developed by the Saudi Data and Artificial Intelligence Authority (SDAIA). It shows the health status of the individual. The study included in the application are: Not proven to have COVID-19, has been in contact with a COVID-19 patient (regardless of the absence of symptoms), has been traveling to countries (A), has been traveling to countries (B); both A and B refer to countries that have high infection rate and require house quarantine after arriving at Saudi Arabia. Only individuals with a healthy status (Not proven to have COVID-19) are allowed in SSDC; otherwise, the security person has the authority to dismiss the individual whether they are learners, faculty, or staff. Based on those two measures, two entry routes were allowed in the center. One is from the healthcare colleges where the security checks the students in their college. The other route is the center's main entrance, where security personnel check each person's temperature and health status.

Another measure is transforming the faculty development program that the Academic Program Department annually conducts in SSDC for PNU faculty involved in simulation activities from onsite training to online lectures. For practical training on operating simulation manikins, the Healthcare Simulation Technology department in SSDC has developed a training program with limited attendance and no more than three faculty per training program. The faculty development program was designed to ensure social distancing and limit infection since training sites are restricted to the simulation theater with a maximum capacity of six participants.

Another change implemented was transforming the learner’s orientation day from onsite lectures and SSDC tours to view the center venues to online lectures. This orientation aimed to introduce the PNU students to simulation education, the SSDC background and services, psychological safety, and physical safety involved in the simulation event. The topics are designed to orient the students about simulation education and give them enough information before signing a confidentiality agreement and fiction agreement issued by SSDC. This agreement stipulates that all events are fictional and that the aim is to learn from the mistakes that occur, and that all attendances will not share information about the event with people who did not attend the event. Before COVID-19, the students used to sign the contract on a hard copy of the agreement. However, after the COVID-19 pandemic broke out, SSDC recommended that simulation coordinators in the healthcare colleges let the students sign the contract on a soft copy. The SSDC simulation educators have focused on ensuring all learners are oriented by asking questions about the agreement and holding a more extended briefing and orientation session before the student starts any simulation event.

All events conducted are closely monitored by the SSDC simulation staff to ensure participants' psychological and physical safety. After the COVID-19 pandemic, close monitoring has become critical to ensure compliance with infection control measures. Faculty were informed that they carry part of the responsibility of ensuring compliance and reporting violations. 

Another measure taken was related to the preparation of medical supplies and equipment for simulation events. Before COVID-19, equipment was shared to limit wasting supplies. However, after the pandemic broke out, it was crucial to ensure that the shared equipment, supplies, and devices would be sanitized. SSDC provided alcohol wipes, alcohol pads, and disinfectant medical spray to sanitize all equipment before sharing them. Hand washing and hand hygiene were enforced on all participants and staff at all times during training.

While most participants in simulation activities have followed the infection control protocol, specific incidences have been reported by simulation educators and SSDC staff. Necessary actions were taken right away according to an action plan that was developed for this purpose. Some of the actions taken against the violated learners who did not follow instructions were verbal warnings and did not allow them to enter the training venues without masks.

Some examples of infection control violations are students not adhering to social distancing rules, students sharing equipment without sanitization, and students not wearing masks during the simulation event. In these types of violations, they are usually given a warning. If repeated, assigned SSDC staff must take the student’s name and ID number to file an incident report to the college dean. This escalation has not occurred because most students comply with first warnings. Some rare and severe violations are students attending simulation events while showing fever, headache, and coughing symptoms, and students showing status in the Tawakalna application other than healthy. In case of severe violations, the student is dismissed from SSDC, and their information is taken to be reported to their college dean.

Data analysis

Statistical Package for the Social Sciences (SPSS) version 21 (IBM Corp., Armonk, NY) was used for data entry and analysis. Qualitative data, including simulation activity type, simulation activity name, undergraduate college name, and undergraduate level, were presented as frequencies and percentages. Numerical data, including participant's evaluation, were presented as means and standard deviations. The evaluation results were divided into two groups based on the time the simulation event was conducted. The first group included evaluation collected before the COVID-19 pandemic, and the second group included evaluation of simulation activities conducted during the COVID-19 pandemic and after implementing infection control precautions. The grouping is done to compare the effect of the infection control precautions on the participants' evaluation of simulation activities. An independent sample T-test was used to compare the evaluation results of the activities conducted before and during the COVID-19 pandemic. A p-value of <0.05 was considered to show a statistically significant difference.

Ethical consideration

Participants' data were kept secure, and only the research team had access to the research data. Data entry on computers did not contain any identification of the respondent. The study was approved by the Princess Nourah University (PNU) Institutional Review Board (IRP), IRP log number was (20-0316).

## Results

After reviewing SSDC records, 837 participants were randomly chosen from different healthcare backgrounds and levels between the 2019 and 2021 academic years. Of the five healthcare colleges that conducted activities in SSDC, 38.1% were from the college of health and rehabilitation sciences, followed by the college of nursing (29.3%), then the college of medicine (24.5%), and finally, the college of pharmacy (8.1%). The college of dentistry did not conduct any activities in the specified period. The majority of the undergraduate students were from levels 4 to level 6 (37.2%). Table 2 shows the characteristics of the study participants.

**Table 2:** Basic characteristics of the study participants

		Event time		Total (n = 837) N (%)
		Before COVID-19 n (%)	During COVID-19 n (%)	
Event Type	Full simulation (FS) (n = 352)	244 (69.3%)	108 (30.7%)	352 (42.1%)
	Procedural simulation (PS) (n= 485)	239 (49.3%)	246 (50.7%)	485 (57.9%)
College	College of Medicine (n = 205)	86 (42.0%)	119 (58.0%)	205 (24.5%)
	College of Health and Rehabilitation Sciences (n = 319)	177 (55.5%)	142 (44.5%)	319 (38.1%)
	College of Pharmacy (n = 68)	49 (72.1%)	19 (27.9%)	68 (8.1%)
	College of Nursing (n = 245)	171 (69.8%)	74 (30.2%)	245 (29.3%)
Participant level	Level 1 - Level 3 (n = 271)	165 (60.9%)	106 (39.1%)	271 (32.4%)
	Level 4 - Level 6 (n = 311)	166 (53.4%)	145 (46.6%)	311 (37.1%)
	Level 7 - Level 9 (n = 132)	69 (52.3%)	63 (47.7%)	132 (15.8%)
	Level 10 - Level 12 (n = 123)	83 (67.5%)	40 (32.5%)	123 (14.7%)

Simulation activities

Due to the COVID-19 pandemic, the number of participants in the simulation activities was less than before the pandemic, which has led to a lower number of evaluations ((n = 354 (40%); n = 483 (60%); respectively)). FS activities were 352 (42.1%) and PS activities were 485 (57.9%). Table 3 shows the frequency of participants’ evaluation of simulation activities included in the study conducted before and during the COVID-19 pandemic. The healthcare colleges vary in the type of activity they run in SSDC.

**Table 3:** Frequency of participant’s evaluation of simulation activities conducted before and during the COVID-19 pandemic

Simulation activity type	Simulation activity name	Before COVID-19 N (%)	During COVID-19 N (%)
Full simulation (FS) (n = 352)	Asthma Exacerbation (Adult Case) (n= 27)	21 (77.8%)	6 (22.2%)
	Asthma Exacerbation (Pediatric Case) (n= 6)	5 (83.3%)	1 (16.7%)
	Burn care (n= 101)	81 (79.4%)	21 (20.6%)
	Preeclampsia (n= 20)	14 (70.0%)	6 (30.0%)
	Status epilepticus (n= 9)	5 (55.6%)	4 (44.4%)
	Heparin Induced Thrombocytopenia (n= 8)	1 (12.5%)	7 (87.5%)
	Post-Partum Hemorrhage (PPH) (n= 24)	20 (83.3%)	4 (16.7%)
	Paralysis (Left Side) -Stroke Case (n= 107)	58 (54.2%)	49 (45.8%)
	Normal Labor and Fetal Monitoring (n= 18)	17 (94.4%)	1 (5.6%)
	Hyperthyroidism (Thyroid Storm Presenting as Gastroenteritis) (n= 26)	20 (76.9%)	6 (23.1%)
	Emergency Low Segment Cesarean Section (n= 6)	3 (50.0%)	3 (50.0%)
Procedural simulation (PS) (n= 485)	Basic Anesthesia Skills: Airway (n= 27)	2 (7.4%)	25 (92.6%)
	Basic Life Support Techniques (n= 245)	161 (65.7%)	84 (34.3%)
	Basic vital signs Practical (n= 13)	2 (15.4%)	11 (84.6%)
	Central Nervous System (CNS) (n= 23)	14 (60.9%)	9 (39.1%)
	Health Education (n= 4)	3 (75.0%)	1 (25.0%)
	Internal Medicine (n= 67)	18 (26.9%)	49 (73.1%)
	Musculoskeletal System (n= 22)	16 (72.7%)	6 (27.3%)
	Patient Care and Management in Diagnostic Imaging (n= 47)	8 (17.0%)	39 (83.0%)
	Pediatric Procedures (n= 13)	6 (46.2%)	7 (53.8%)
	Surgery (n= 14)	6 (42.9%)	8 (57.1%)
	Total Hip Replacement (n= 10)	3 (30.0%)	7 (70.0%)

COVID-19 infection control protocol

**Figure 2:**
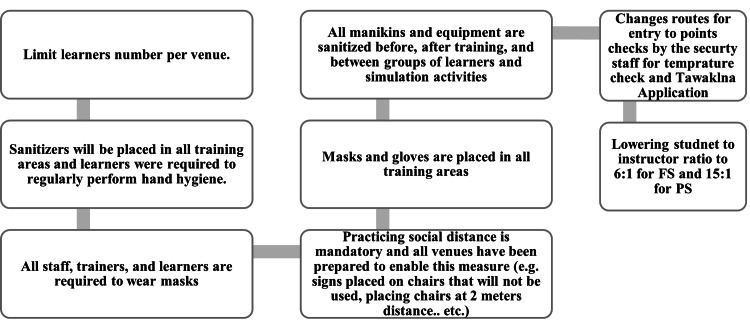
Examples of measures taken according to the SSDC infection control protocol SSDC: Simulation and Skills Development Center

Simulation educators from the department and SSDC staff involved in the education activities implemented the protocol in the center, and it was also reported to all healthcare colleges. Figure 2 shows some examples of the infection control measures followed while conducting simulation activities in SSDC.

Effect of the COVID-19 infection control protocol on participants' evaluation

The learner’s evaluation for all simulation activities was divided into two groups. The first group participated in simulation activities before the COVID-19 infection control protocol. The second group included learners’ evaluations of simulation activities conducted during COVID-19 and after implementing the infection control protocol. After performing a T-test to compare the mean of evaluation results, there was a significant difference in evaluation results of FS and PS activities before and after implementing the COVID-19 infection control protocol (p-value < 0.001). Table 4 shows the mean difference between simulation activities conducted before the COVID-19 pandemic and after implementing the COVID-19 infection control protocol.

**Table 4:** The difference in mean between the participants’ evaluation of simulation activities conducted before and during the COVID-19 pandemic * p-value below <0.05 shows a significant difference in mean

Event type	Group	n (%)	Participant evaluation (Mean ± SD)	p-value
Evaluation of Full simulation (FS) (n = 352) (Total score = 50)	Before COVID-19 pandemic	244 (69.3%)	40.61 ± 12.34	< 0.001^*^
	During COVID-19 pandemic	108 (30.7%)	46.45 ± 6.56	
Evaluation of Procedural simulation (PS) (Total score = 40) (n= 485)	Before COVID-19 pandemic	239 (49.3%)	27.82 ± 11.63	< 0.001^*^
	During COVID-19 pandemic	246 (50.7%)	36.85 ± 5.74	

After analyzing the evaluation results among the colleges, it was shown that the evaluation results of the participants from the college of pharmacy were not significantly different when comparing PS evaluations before and during the COVID-19 pandemic (p-value= 0.157). Table 5 shows the difference in mean between participants’ evaluation of simulation activities conducted before and during the COVID-19 pandemic among healthcare colleges.

**Table 5:** The difference in mean between participants’ evaluation of simulation activities conducted before and during the COVID-19 pandemic among healthcare colleges *Cannot be computed due to missing data **p-value below <0.05 shows a significant difference in mean

Event type	College	Before COVID-19 (Mean ± SD)	During COVID-19 (Mean ± SD)	p-value
FS	College of Medicine	41. 04 ± 14.03	49.37 ± 1.61	0.008^**^
	College of Health and Rehabilitation Sciences	NA^*^	NA^*^	NA^*^
	College of Pharmacy	40.98 ± 11.68	45.32 ± 9.84	0.157
	College of Nursing	40.44 ± 12.35	46.59 ± 6.19	<0.001^**^
PS	College of Medicine	32.18 ± 9.74	37.34 ± 4.829	<0.001^**^
	College of Health and Rehabilitation Sciences	26.29 ± 11.88	36.53 ± 6.36	<0.001^**^
	College of Pharmacy	NA^*^	NA^*^	NA^*^
	College of Nursing	36.00 ± 3.16	NA^*^	NA^*^

## Discussion

This study aimed to investigate the evaluation of simulation events among healthcare learners attending an onsite educational activity during the COVID 19 pandemic and under infection control protocol. The study found a significant difference in the students’ evaluation of simulation training after implementing COVID-19 precautions. The measures enforced by the SSDC didn’t negatively impact the student evaluation in FS and PS activities.

The traditional healthcare educational methodologies have been changed profoundly due to COVID 19. This has resulted in conducting nearly all academic activities on a virtual basis to minimize the in-person interaction between learners and mitigate the risk of spreading the virus. This transformation from the onsite to the virtual environment shortened the volume of hands-on and onsite healthcare educational activities [15]. The student perception of the virtual training has been positive, but they still need simulation training. In the Ferrara et al. study, the collected students’ perception of the recent changes done in the ophthalmology course from 32 countries using an online survey [16]; of the 504 responses, 55.2% described the impact of COVID-19 as severe and 86.9% has agreed that simulation-training is helpful for their training along with web-based training.

While virtual education has shown that it is a reliable education methodology, it has some drawbacks. The absence of physical attendance causes difficulty in training students in complex subjects [17]. Additionally, teachers and faculty members have pointed out that the shift in educational methodologies requires adequate training and guidance to deliver educational objectives using virtual education methodology efficiently [17].

The COVID-9 quarantine and the socioeconomic and safety challenges from the pandemic have impeded in-person training and the ability of educational institutions to conduct simulation training for the duration of the quarantine [18-19]. After the students return to onsite training, their appreciation of the activities has increased. They have started considering the role of teachers and the efficacy of simulation training on skills acquisition [17,19-20].

Therefore, it is critical to establish an infection control protocol to be implemented while conducting simulation-based training for undergraduate students is critical. SSDC took the initiative and implemented an infection control protocol during the COVID-19 pandemic to accept bookings on healthcare training. The attempt to implement the protocol was to ensure learners’ psychological status and promote safety and responsibility among healthcare learners. Different measures must be taken to ensure that infection control is possible to implement, and acquiring support from the center staff was crucial to ensure implementation. Additionally, addressing critical changes in the process of preparing and conducting simulation events must be done to limit the spread of infection among participants and assure them that their environment is safe to the best of their abilities. While reporting incidents was done without an electronic system to track and quantify them, the higher management must offer instructions of how violations should be managed and provide full support for the staff to enable them to implement the protocol. Some challenges face the implementation of infection control protocol, such as securing checkpoints, providing sanitization supplies, providing large venues to conduct activities and ensure social distancing, and ensuring enough staff to monitor simulation activities.

The results of this study proved that the infection control protocol does not negatively affect the students’ evaluation of simulation training. The study has shown that the activities conducted after COVID-19 have higher perception and attitude scores. The differences found between the results were due to several factors that played a significant role in enhancing the outcomes of the educational activity. Those factors include psychological readiness, limiting the attendance numbers, which has changed the faculty to student ratios, and, therefore, the students were more focused and attentive. This study is the first to explore the impact of conducting simulation training under the full infection control protocol; however, it has limitations. Matching students across the study period was not possible due to the process of collecting evaluation. Therefore, only students' affiliation, level, and event type can be matched.

Additionally, the study pool was all-female since PNU is a women's college. Another limitation was the lack of reporting systems for implementing the COVID-19 protocol and incidence reports. Another limitation was that the data were taken from one center.

## Conclusions

The study showed that conducting onsite simulation activities under the infection control protocol does not negatively impact female undergraduate evaluation of simulation events. It is recommended to implement an infection control protocol designed to ensure physical and psychological safety among healthcare learners in educational entities and institutions to tackle challenges during the COVID 19 pandemic. Further studies and investigations on the COVID-19 psychological effects on students attending onsite training and the performance level of faculty members on teaching are needed in the future.
